# An Autoantigen Atlas From Human Lung HFL1 Cells Offers Clues to Neurological and Diverse Autoimmune Manifestations of COVID-19

**DOI:** 10.3389/fimmu.2022.831849

**Published:** 2022-03-24

**Authors:** Julia Y. Wang, Wei Zhang, Victor B. Roehrl, Michael W. Roehrl, Michael H. Roehrl

**Affiliations:** ^1^ Curandis, New York, NY, United States; ^2^ Department of Gastroenterology, Affiliated Hospital of Guizhou Medical University, Guiyang, China; ^3^ Department of Pathology and Laboratory Medicine, Memorial Sloan Kettering Cancer Center, New York, NY, United States; ^4^ Human Oncology and Pathogenesis Program, Memorial Sloan Kettering Cancer Center, New York, NY, United States

**Keywords:** COVID-19, SARS-CoV-2, autoantigens, autoantibodies, dermatan sulfate, autoimmunity

## Abstract

COVID-19 is accompanied by a myriad of both transient and long-lasting autoimmune responses. Dermatan sulfate (DS), a glycosaminoglycan crucial for wound healing, has unique affinity for autoantigens (autoAgs) from apoptotic cells. DS-autoAg complexes are capable of stimulating autoreactive B cells and autoantibody production. We used DS-affinity proteomics to define the autoantigen-ome of lung fibroblasts and bioinformatics analyses to study the relationship between autoantigenic proteins and COVID-induced alterations. Using DS-affinity, we identified an autoantigen-ome of 408 proteins from human HFL1 cells, at least 231 of which are known autoAgs. Comparing with available COVID data, 352 proteins of the autoantigen-ome have thus far been found to be altered at protein or RNA levels in SARS-CoV-2 infection, 210 of which are known autoAgs. The COVID-altered proteins are significantly associated with RNA metabolism, translation, vesicles and vesicle transport, cell death, supramolecular fibrils, cytoskeleton, extracellular matrix, and interleukin signaling. They offer clues to neurological problems, fibrosis, smooth muscle dysfunction, and thrombosis. In particular, 150 altered proteins are related to the nervous system, including axon, myelin sheath, neuron projection, neuronal cell body, and olfactory bulb. An association with the melanosome is also identified. The findings from our study illustrate a connection between COVID infection and autoimmunity. The vast number of COVID-altered proteins with high intrinsic propensity to become autoAgs offers an explanation for the diverse autoimmune complications in COVID patients. The variety of autoAgs related to mRNA metabolism, translation, and vesicles suggests a need for long-term monitoring of autoimmunity in COVID. The COVID autoantigen atlas we are establishing provides a detailed molecular map for further investigation of autoimmune sequelae of the pandemic, such as “long COVID” syndrome.

**Summary Sentence:**

An autoantigen-ome by dermatan sulfate affinity from human lung HFL1 cells may explain neurological and autoimmune manifestations of COVID-19.

## Introduction

The emergence of the novel coronavirus SARS-CoV-2 has dragged the world into a prolonged pandemic. Aside from the intensively studied ACE2, heparan sulfate is another crucial entry receptor for coronaviruses (1). Dermatan sulfate (DS), structurally and functionally similar to heparan sulfate and heparin, belongs to the glycosaminoglycan family. Many viruses, including Ebola, Vaccinia, Zika, Dengue, and Hepatitis C viruses, have been shown to interact with glycosaminoglycans (2–5). These polyanionic polysaccharides consist of disaccharide repeating units of amino sugars and uronic acids with varying degrees of sulfation. Glycosaminoglycans are major components of the extracellular matrix and basement membrane, act as a filler between cells and tissue fibers and have numerous biological functions.

DS is most abundant in the skin but is also found in lungs, blood vessels, heart valves, and tendons. DS plays important roles in cell death, wound healing, and tissue repair. In human wound fluid, DS is the most abundant glycosaminoglycan (6). Its biosynthesis is increased by fibroblasts, epithelial cells, and capillary endothelial cells in wounded skin, mucosal ulcers, and inflammation-associated angiogenesis (7–9). Its molecular size also changes during wound healing, with elongated DS polymers packing along thin collagen fibrils in wounded skin (10). After tissue injury, fibroblasts require DS to migrate from the stroma surrounding the injury into the fibrin-laden wound to facilitate granulation tissue formation and wound healing (11).

DS is also a key molecule in autoimmunity, as we have discovered (12–16). DS is the most potent among glycosaminoglycans in stimulating autoreactive B1 cells and autoantibody production (12, 13). DS has a peculiar affinity to apoptotic cells and their released autoantigens (autoAgs), and macromolecular autoAg-DS affinity complexes are capable of engaging autoBCRs in a dual signaling event to activate B1 cells (13, 14). Recently, we also found that DS may steer autoreactive B1 cell fate at the pre-B stage by regulating the immunoglobulin heavy chain of the precursor BCR (17). Our studies illustrate a unifying property of autoAgs, i.e., self-molecules with DS affinity have a high propensity to become autoAgs, which explains how seemingly unrelated self-molecules can all induce humoral autoimmunity *via* similar immunological signaling events. In support of our hypothesis and by using DS affinity, we have cataloged hundreds of classic and novel autoAgs (14–16, 18).

A diverse spectrum of autoimmune symptoms has been observed in COVID-19 patients, including autoimmune cytopenia, multisystem inflammatory syndrome in children, immune-mediated neurological syndromes, Guillain-Barré syndrome, connective tissue disease-associated interstitial lung disease, antiphospholipid syndrome, autoimmune hemolytic anemia, autoimmune encephalitis, systemic lupus erythematosus, optic neuritis and myelitis, and acquired hemophilia (19–26). Many autoantibodies have been identified in COVID patients, including ANA (antinuclear antibody), ENA (extractable nuclear antigen), ANCA (anti-neutrophil cytoplasmic antibody), lupus anticoagulant, antiphospholipid, anti-IFN, anti-myelin oligodendrocyte glycoprotein, and anti-heparin-PF4 complex (19–27).

To understand autoimmune sequelae of COVID, we aimed to establish a COVID autoantigen atlas that will serve as a molecular map to guide ongoing research into autoimmune sequelae of COVID (such as “long COVID” syndrome) and vaccine evaluation. Acute COVID leads to significant acute inflammatory lung injury and histologic remodeling that involves marked lung fibroblast activation and cell turnover (28). In this study, we identified an autoantigen-ome of 408 proteins from human fetal lung fibroblast HFL1 cells by DS-affinity fractionation and protein sequencing, with at least 231 being known autoAgs. We then compared these with currently available data from SARS-CoV-2-infected patients and cells (as of 12/14/2020 in Coronascape) (29–49). Remarkably, 352 (86.3%) of these proteins have been found to be altered (up- or down-regulated) at protein and/or RNA expression levels, and 210 of the COVID-altered proteins are known autoAgs in a great variety of autoimmune diseases and cancers. The COVID-altered proteins reveal intricate host responses to the viral infection and point to close associations with diverse disease manifestations of COVID-19.

## Results and Discussion

### An Autoantigen-Ome of 408 Proteins With DS-Affinity From HFL1 Cells

Proteins extracted from HFL1 cells were fractionated with DS-affinity resins. The DS-binding fraction eluting with 0.5 M NaCl yielded 306 proteins by mass spectrometry sequencing, corresponding to proteins with medium-to-strong DS affinity. The fraction eluting with 1.0 M NaCl yielded 121 proteins, corresponding to proteins with very strong DS affinity. After excluding redundancies, a total of 408 unique proteins were obtained (**Table 1**). To verify how many of these proteins are known autoAgs, we conducted an extensive literature search for autoantibodies specific for each protein. Remarkably, at least 231 (57%) of our DS-affinity proteins already have known associated specific autoantibodies in various diseases and are thus confirmed autoAgs, corresponding to 61% of proteins with very strong DS affinity and 54% of proteins with medium-to-strong DS affinity (see references in **Table 1**).

**Table 1:** DS-affinity enriched autoantigen-ome from human HFL1 cells.

# Pep.	Gene	Protein	COVID		DS-affinity		Ref.
			Up	Down	1.0 M	0.5 M	
5	A2M	Alpha-2-macroglobulin		D		+	(50)
5	AARS	Alanyl-tRNA synthetase, cytoplasmic	U	D		+	(51)
10	ACTA2	Actin, aortic smooth muscle	U	D		+	(52)
8	ACTB	Actin, cytoplasmic	U	D		+	(53)
6	ACTBL2	Beta-actin-like protein	U	D		+	
17	ACTN1	Alpha-actinin-1	U	D		+	(54)
6	ACTN4	Alpha-actinin-4	U	D		+	(52)
3	AFP	Alpha-fetoprotein		D		+	(55)
5	AHNAK	Neuroblast differentiation-associated protein	U	D		+	(56)
10	ALB	Putative uncharacterized protein albumin	U	D		+	(57)
3	ALPP	Alkaline phosphatase, placental type precursor			+		(58)
6	ANP32A	Acidic leucine-rich nuclear phosphoprotein 32 member A	U	D	+		
11	ANP32B	Acidic nuclear phosphoprotein 32 family member B		D	+		
3	ANP32C	Acidic nuclear phosphoprotein 32 family member C			+		
3	ANP32E	Acidic nuclear phosphoprotein 32 family member E	U	D	+		
2	ANXA2	Annexin A2	U	D	+		(59)
7	ANXA2P2	Putative annexin A2-like protein, ANX2L2, LPC2B	U	D		+	
7	ANXA5	Annexin A5	U	D		+	(60)
33	ANXA6	Annexin VI	U	D		+	(61)
2	AP1B1	AP-1 complex subunit beta-1				+	
2	AP3B1	AP-3 complex subunit beta-1	U		+		
2	AP3B2	AP-3 complex subunit beta-2			+		(62)
3	AP3D1	AP-3 complex subunit delta-1	U	D	+		
3	APOA1	Apolipoprotein A-I		D		+	(63)
2	APOD	Apolipoprotein D	U	D		+	
2	ARCN1	Coatomer delta, Archain vesicle transport protein 1		D		+	
4	ARF1	ADP-ribosylation factor				+	
2	ARHGAP1	Rho-GTPase-activating protein	U			+	
4	ARHGDIA	Rho GDP-dissociation inhibitor 1	U	D		+	
9	ATP5B	ATP synthase subunit beta, ATP5F1B	U	D		+	(64)
3	BCAT1	Branched chain amino acid aminotransferase	U			+	
2	BCCIP	BRCA2 and CDKN1A-interacting protein				+	
2	BGN	Biglycan			+		(65)
2	BSG	Basigin, CD147		D	+		(66)
2	BZW2	Basic leucine zipper and W2 domains 2				+	
7	C1QBP	Complement C1q-binding protein		D	+		(67)
7	CALD1	Caldesmon		D		+	
8	CALM1	CALM3; CALM2 Calmodulin	U	D		+	(21)
16	CALR	Calreticulin	U	D		+	(68)
2	CALU	Calumenin	U	D		+	(69)
3	CANX	Calnexin	U	D	+		(70)
9	CAP1	Adenylyl cyclase-associated protein	U	D		+	
7	CAPN1	Calpain-1 catalytic subunit				+	
5	CAPN2	Calpain-2 catalytic subunit	U	D		+	(21)
3	CAPNS1	Calpain small subunit				+	
2	CAPZA1	F-actin-capping protein subunit alpha-1		D	+		(71)
3	CAPZB	F-actin-capping protein subunit beta		D		+	(72)
8	CAVIN1	Caveolae-associated protein 1, PTRF	U	D		+	
3	CBX1	Chromobox protein homolog	U			+	(73)
3	CCDC6	Coiled-coil domain-containing protein	U	D		+	(74)
3	CCT2	T-complex protein 1 subunit beta		D		+	
3	CCT8	T-complex protein 1 subunit theta	U	D		+	(75)
4	CD248	Endosialin		D		+	
5	CDC37	Hsp90 co-chaperone Cdc37	U	D		+	
4	CKAP4	Cytoskeleton-associated protein 4, P63	U	D	+		(76)
8	CKB	Creatine kinase B-type	U	D		+	(77)
7	CLIC1	Chloride intracellular channel protein	U	D		+	
2	CLIC4	Chloride intracellular channel protein	U	D		+	
14	CLTC	Clathrin heavy chain 1	U	D	+		(14)
3	CLTCL1	Clathrin heavy chain 2			+		
3	CNPY2	Protein canopy homolog		D		+	
13	COL12A1	Collagen type XII alpha-1 chain	U	D		+	
45	COL1A1	Collagen type I alpha-1 chain	U	D		+	(78)
37	COL1A2	Collagen type I alpha-2 chain		D		+	(79)
2	COL2A1	Collagen type II alpha-1 chain	U			+	(80)
12	COL3A1	Collagen type III alpha-1 chain				+	(81)
3	COL5A1	Collagen type V alpha 1	U			+	(82)
6	COL6A1	Collagen type VI alpha-1 chain		D		+	(83)
4	COL6A2	Collagen type VI alpha-2 chain		D		+	
29	COL6A3	Collagen type VI alpha-3 chain		D		+	
2	COPA	Coatomer subunit alpha	U	D	+		(84)
2	COPB1	Coatomer subunit beta		D	+		(85)
5	COPB2	Coatomer subunit beta’	U		+		(86)
2	COPZ1	Coatomer subunit zeta-1		D		+	
3	CORO1C	Coronin-1C				+	
4	CRK	Proto-oncogene C-crk	U	D		+	
5	CRTAP	Cartilage-associated protein, P3H5		D	+		
4	CSPG4	Chondroitin sulfate proteoglycan 4		D	+		(87)
3	CTSB	Cathepsin B, APP secretase	U	D		+	
2	CTSD	Cathepsin D	U	D		+	(88)
2	CUTA	CutA divalent cation tolerance homolog	U	D		+	
2	DBN1	Drebrin 1	U	D		+	(89)
3	DCN	Decorin		D	+		(90)
2	DCTN1	Dynactin subunit 1, 150 KDa Dynein-associated protein		D	+		(91)
5	DCTN2	Dynactin subunit 2				+	
12	DDB1	DNA damage-binding protein 1	U	D		+	(14)
2	DDX39	ATP-dependent RNA helicase DDX39A	U	D		+	
5	DDX39B	Spliceosome RNA helicase BAT1		D		+	
5	DHX15	ATP-dependent RNA helicase #46		D	+		
5	DHX9	ATP-dependent RNA helicase A			+		(92)
5	DIABLO	Diablo, IAP (Inihibitor of apoptosis protein)-binding	U			+	
2	DKC1	H/ACA ribonucleoprotein complex subunit DKC1	U	D	+		
2	DLST	Dihydrolipoyllysine-residue succinyltransferase component of 2- oxoglutarate dehydrogenase complex		D		+	(93)
2	DNAJB11	DnaJ (Hsp40) homolog subfamily B member 11	U			+	(94)
2	DPP3	Dipeptidyl-peptidase 3		D		+	
3	DPYSL2	Dihydropyrimidinase-related protein	U	D		+	(95)
3	DRG1	Developmentally-regulated GTP-binding protein		D		+	
5	DYNC1H1	Dynein cytoplasmic 1 heavy chain 1			+		
2	DYNC1I2	Dynein cytoplasmic 1 intermediate chain 2			+		
2	EEF1A1	Elongation factor 1-alph 1	U	D		+	(96)
3	EEF1A2	Elongation factor 1-alpha 2	U			+	(97)
2	EEF1B2	Elongation factor 1-beta 2		D		+	
5	EEF1D	Elongation factor 1-delta		D		+	
10	EEF1G	Elongation factor 1-gamma	U	D		+	
14	EEF2	Elongation factor 2	U	D		+	(98)
6	EFTUD2	116 kDa U5 snRNP component, SNRP116		D	+		(99)
4	EHD2	EH domain-containing protein 2	U	D		+	
3	EIF2S1	Eukaryotic translation initiation factor 2 subunit 1, EIF2A				+	(100)
10	EIF3A	Eukaryotic translation initiation factor 3 subunit A	U	D	+		(101)
9	EIF3B	Eukaryotic translation initiation factor 3 subunit B	U	D	+		
3	EIF3CL	Eukaryotic translation initiation factor 3 subunit C-like protein		D	+		
5	EIF3E	Eukaryotic translation initiation factor 3 subunit E	U	D	+		(102)
2	EIF3F	Eukaryotic translation initiation factor 3 subunit F	U	D	+		
2	EIF3G	Eukaryotic translation initiation factor 3 subunit G				+	
6	EIF3L	EIF3, subunit E interacting protein		D	+		
11	EIF4A1	Eukaryotic initiation factor 4A-1, DDX2A	U	D		+	
2	EIF4A3	Eukaryotic initiation factor 4A-III, DDX48				+	(103)
4	EIF4G1	Eukaryotic translation initiation factor 4 gamma 1	U	D		+	
2	EIF4G2	Eukaryotic translation initiation factor 4 gamma 2		D		+	
4	EIF5A	Eukaryotic translation initiation factor 5A-1	U	D		+	
2	EIF5A2	Eukaryotic translation initiation factor 5A-2		D		+	
3	EIF6	Eukaryotic translation initiation factor 6	U			+	
4	ELAVL1	ELAV-like protein		D		+	(104)
2	ELOB	Transcription elongation factor B, TCEB2	U	D		+	
2	ENO1	Alpha-enolase	U	D		+	(105)
7	ENO2	Gamma-enolase	U	D		+	(106)
2	ENOPH1	Enolase-phosphatase E1	U			+	
2	EPRS	Bifunctional aminoacyl-tRNA synthetase, EPRS1	U		+		(107)
6	ERP44	Endoplasmic reticulum resident protein ERp44				+	(108)
2	EWSR1	EWS RNA-binding protein	U			+	
2	FAF1	FAS-associated factor 1	U			+	
4	FAM62A	Extended synaptotagmin-1, ESYT1			+		(109)
2	FASN	Fatty acid synthase	U	D		+	(110)
3	FBLN1	Fibulin 1	U	D		+	(111)
8	FKBP10	FK506-binding protein 10				+	
4	FKBP9	FK506-binding protein 9		D		+	
43	FLNA	Filamin-A	U	D		+	(112)
8	FLNB	Filamin-B	U			+	(14)
24	FLNC	Filamin-C	U	D		+	(113)
23	FN1	Fibronectin	U	D		+	(114)
3	FSTL1	Follistatin-related protein	U	D		+	(115)
2	FTH1	Ferritin heavy chain	U	D		+	(115)
2	G6PD	Glucose-6-phosphate 1-dehydrogenase	U	D		+	
15	GANAB	Neutral alpha-glucosidase AB		D		+	(116)
2	GAPDH	Glyceraldehyde-3-phosphate dehydrogenase	U	D		+	(117)
2	GAR1	H/ACA ribonucleoprotein complex subunit 1			+		
2	GDI1	Rab GDP dissociation inhibitor alpha	U	D		+	(118)
2	GDI2	Rab GDP dissociation inhibitor beta	U	D		+	(119)
2	GLRX3	Glutaredoxin 3, Thioredoxin-like 2		D		+	(120)
2	GMFB	Glia maturation factor, beta	U			+	
5	GPC1	Glypican-1		D	+		
16	GSN	Gelsolin	U	D		+	(121)
4	GTF2I	General transcription factor II-I (GTF2IP4)	U	D		+	
2	H2AFV	Histone H2A.V, H2AZ2		D	+		(122)
4	H2AFY2	Histone marcoH2A1, MAROH2A1	U		+		(123)
2	HARS	Histidyl-tRNA synthetase, cytoplasmic				+	(21)
3	HDGF	Hepatoma-derived growth factor	U	D		+	(124)
2	HDLBP	Vigilin, High density lipoprotein binding protein	U	D		+	
2	HEBP2	Heme-binding protein 2	U			+	
5	HEXB	Beta-hexosaminidase subunit beta		D		+	
4	HIST1H1B	Histone H1.5, H1-5	U	D		+	(125)
4	HIST1H1C	Histone H1.2, H1-2	U	D	+		(125)
2	HIST1H2BL	Histone H2B type 1-L, H2BC13	U	D	+		(126)
9	HIST1H4J	Histone H4, H4C1			+		(127)
11	HIST2H2BE	Histone H2B type 2-E, H2BC21	U	D	+		(128)
3	HIST2H3D	Histone H3.2, HIST2H3A, HIST2H3C, H3C13			+		(129)
4	HMGB1L1	High mobility group box 1 pseudogene 1, HMGB1P1				+	(130)
2	HNRNPA1	U1 ribonucleoprotein A1	U	D		+	(131)
5	HNRNPA2B1	Putative uncharacterized protein HNRNPA2B1	U	D		+	(132)
2	HNRNPA3	Heterogeneous nuclear ribonucleoprotein A3	U	D		+	(133)
2	HNRNPC	Heterogeneous nuclear ribonucleoproteins C1/C2	U	D	+		(134)
7	HNRNPCL1	Heterogeneous nuclear ribonucleoprotein C-like 1				+	
2	HNRNPD	Heterogeneous nuclear ribonucleoprotein D, AUF1				+	(135)
3	HNRNPDL	Heterogeneous nuclear ribonucleoprotein D-like	U	D		+	(136)
5	HNRNPF	Heterogeneous nuclear ribonucleoprotein F		D		+	(137)
2	HNRNPH1	Heterogeneous nuclear ribonucleoprotein H1	U	D		+	(137)
2	HNRNPH3	Heterogeneous nuclear ribonucleoprotein H3	U	D		+	
9	HNRNPK	Heterogeneous nuclear ribonucleoprotein K	U			+	(138)
7	HNRNPR	Heterogeneous nuclear ribonucleoprotein R	U	D		+	(139)
5	HNRNPU	Heterogeneous nuclear ribonucleoprotein U	U	D		+	
3	HNRNPUL1	HnRNP U-like protein 1	U	D	+		
11	HSP90AA1	Heat shock 90kDa protein 1, alpha isoform	U	D		+	(140)
3	HSP90AA2	Putative heat shock protein HSP 90-alpha A				+	(141)
11	HSP90AB1	Heat shock protein HSP 90-beta	U	D		+	(142)
31	HSP90B1	Endoplasmin	U	D		+	(143)
3	HSPA1A	HSPA1B Heat shock 70 kDa protein 1A	U	D		+	
2	HSPA1L	Heat shock 70 kDa protein 1-like				+	(144)
2	HSPA4	Heat shock 70 kDa protein 4	U	D		+	
28	HSPA5	Endoplasmic reticulum chaperone BiP, GRP78	U	D		+	(145)
27	HSPA8	Heat shock cognate 71 kDa protein	U	D		+	(146)
8	HSPA9	Stress-70 protein, mitochondrial	U	D		+	(146)
7	HSPB1	Heat shock protein beta-1	U	D		+	(147)
2	HSPD1	60 kDa heat shock protein, mitochondrial	U	D		+	
3	HSPG2	Basement membrane heparan sulfate proteoglycan	U	D	+		(148)
2	HTATSF1	HIV Tat-specific factor 1		D	+		
7	HYOU1	Hypoxia up-regulated protein	U			+	
2	IGBP1	Immunoglobulin-binding protein 1	U	D		+	
7	ILF2	Interleukin enhancer-binding factor	U			+	(149)
2	ILF3	Interleukin enhancer-binding factor 3	U			+	(149)
13	IQGAP1	Ras GTPase-activating-like protein IQGAP1	U			+	(150)
2	IRGQ	Immunity-related GTPase family Q protein	U	D		+	
4	ITGB1	Integrin beta-1	U	D	+		
4	KARS	Lysyl-tRNA synthetase				+	(107)
2	KPNA3	Importin subunit alpha-4			+		
8	KPNB1	Importin subunit beta-1			+		(151)
10	KTN1	Kinectin	U			+	(152)
7	LAMB1	Laminin subunit beta-1		D		+	(153)
5	LAMC1	Laminin subunit gamma-1	U	D		+	(154)
3	LCP1	Plastin-2	U	D		+	(155)
5	LGALS1	Galectin-1	U	D		+	(156)
23	LMNA	Isoform A of Lamin-A/C	U	D		+	(157)
3	LMNB1	Lamin-B1	U	D		+	(158)
7	LMNB2	Lamin-B2	U	D	+		(159)
2	LRPPRC	Leucine-rich PPR motif-containing protein		D		+	(160)
2	LSM2	U6 snRNA-associated Sm-like protein LSm2	U			+	
2	LSM6	U6 snRNA-associated Sm-like protein LSm6	U			+	
2	MAGOHB	Protein mago nashi homolog	U	D		+	
3	MANBA	Beta-mannosidase		D		+	
3	MAP1B	Microtubule-associated protein 1B	U	D		+	(161)
6	MAPRE1	Microtubule-associated protein RP/EB family member				+	
10	MOV10	Putative helicase, Moloney leukemia virus 10 protein	U	D	+		
3	MSN	Moesin	U			+	(162)
21	MVP	Major vault protein	U	D	+		(163)
4	MXRA5	Matrix-remodeling-associated protein 5		D	+		(163)
2	MYH10	Myosin-10	U	D	+		(164)
43	MYH9	Myosin-9	U	D	+		(164)
3	MYL6	Myosin light chain 6	U			+	
4	MYLK	Myosin light chain kinase, smooth muscle	U	D		+	
3	MYO1C	Unconventional myosin-Ic		D	+		(165)
2	NACA	Nascent polypeptide associated complex subunit alpha	U	D		+	(166)
3	NAP1L1	Nucleosome assembly protein 1-like 1	U	D	+		
3	NAP1L4	Nucleosome assembly protein 1-like 4	U	D	+		
2	NASP	Nuclear autoantigenic sperm protein	U	D		+	(167)
11	NCL	Nucleolin	U	D	+		(168)
2	NES	Nestin	U	D		+	
2	NEU1	Sialidase-1	U	D		+	(169)
3	NEXN	Nexilin F-actin binding protein	U	D		+	
2	NFU1	HIRA interacting protein 5				+	
3	NME1	Nucleoside diphosphate kinase A, RMRP	U	D		+	(170)
2	NMT1	Glycylpeptide N-tetradecanoyltransferase 1				+	(171)
2	NMT2	Glycylpeptide N-tetradecanoyltransferase 2		D		+	
4	NPEPPS	Puromycin-sensitive aminopeptidase				+	
7	NPM1	Nucleophosmin	U	D		+	(172)
5	NUDC	Nuclear distribution C, Dynein complex regulator		D		+	
3	NUDT21	Cleavage and polyadenylation specificity factor 5		D		+	
2	NUDT5	Nudix hydrolase 5		D		+	
3	NUMA1	Nuclear mitotic apparatus protein 1	U	D		+	(173)
5	P3H1	Basement membrane chondroitin sulfate proteoglycan	U			+	
2	P3H3	Prolyl 3-hydroxylase 3, LEPREL2		D	+		
2	P3H4	ER protein SC65, nucleolar autoantigen No55			+		(174)
2	P4HA2	Prolyl 4-hydroxylase subunit alpha-2		D		+	
18	P4HB	Protein disulfide-isomerase	U	D		+	(175)
4	PA2G4	Proliferation-associated protein 2G4	U	D		+	
19	PABPC1	Poly(A)-binding protein 1		D	+		(176)
7	PABPC4	Poly(A)-binding protein 4, APP1		D	+		(177)
3	PARVA	Alpha-parvin	U			+	
4	PCNA	Proliferating cell nuclear antigen	U	D		+	(178)
17	PDIA3	Protein disulfide-isomerase A3	U	D		+	(179)
34	PDIA4	Protein disulfide-isomerase A4	U	D		+	
9	PDIA6	Protein disulfide-isomerase A6	U	D		+	
3	PFDN2	Prefoldin subunit 2	U			+	(180)
8	PFN1	Profilin-1	U	D		+	(181)
2	PFN2	Profilin-2	U			+	(182)
91	PLEC	Plectin-1, PLEC1	U	D		+	(183)
5	PLOD1	Procollagen-lysine, 2-oxoglutarate 5-dioxygenase 1		D		+	
5	PLOD3	Multifunctional procollagen lysine hydroxylase and glycosyltransferase LH3				+	
6	PLS3	Plastin-3	U	D		+	
10	PPIB	Peptidyl-prolyl cis-trans isomerase	U	D		+	(184)
4	PRDX3	Thioredoxin-dependent peroxide reductase	U	D		+	(185)
3	PRDX4	Peroxiredoxin-4	U	D		+	(186)
2	PRKAR2A	Protein kinase CAMP-dependent type II regulatory alpha	U			+	
2	PRKCDBP	Protein kinase C delta-binding protein				+	
11	PRKCSH	Protein kinase C substrate 80K-H		D		+	
5	PRKDC	DNA-dependent protein kinase catalytic subunit	U	D	+		(187)
4	PRMT1	Protein arginine N-methyltransferase 1		D		+	
24	PRPF8	Pre-mRNA-processing-splicing factor 8	U	D	+		(14)
2	PSAP	Proactivator polypeptide, Prosaposin	U	D		+	
5	PSMA3	Proteasome subunit alpha type-3, C8	U	D		+	(188)
4	PSMA4	Proteasome subunit alpha type-4, C9	U			+	(189)
4	PSMA5	Proteasome subunit alpha type-5	U			+	(190)
6	PSMA6	Proteasome subunit alpha type-6	U	D		+	
6	PSMA7	Proteasome subunit alpha type-7	U	D		+	(191)
5	PSMB1	Proteasome subunit beta type-1				+	(192)
2	PSMB3	Proteasome subunit beta type-3		D		+	(188)
7	PSMB4	Proteasome subunit beta type-4				+	
3	PSMB6	Proteasome subunit beta type-6		D		+	
5	PSMB7	Proteasome subunit beta type-7		D		+	
2	PSMD1	26S proteasome non-ATPase regulatory subunit 1	U		+		
2	PSMD12	26S proteasome non-ATPase regulatory subunit 12		D	+		
3	PSMD13	Proteasome 26S non-ATPase subunit 13		D		+	(193)
9	PSMD6	26S proteasome non-ATPase regulatory subunit 6				+	
2	PSMD7	26S proteasome non-ATPase regulatory subunit 7	U			+	
6	PTBP1	Polypyrimidine tract-binding protein, hnRNP I	U	D		+	(194)
2	PTCD3	Pentatricopeptide repeat domain 3, MRPS39			+		
2	PUF60	Poly(U)-binding-splicing factor PUF60	U			+	(195)
2	PZP	Pregnancy zone protein, alpha-2-macroglobulin like		D		+	(196)
4	QARS	Glutaminyl-tRNA synthetase			+		(107)
3	RAB1A	Ras-related protein Rab-1A		D	+		
3	RAB7A	Ras-related protein Rab-7a	U	D		+	
3	RAD23A	UV excision repair protein RAD23 homolog A		D		+	(197)
5	RAD23B	UV excision repair protein RAD23 homolog B	U	D		+	(197)
6	RALY	RNA binding protein, autoantigen p542	U	D	+		(198)
5	RBBP4	Chromosome assembly factor 1 subunit C		D	+		(199)
2	RBM3	Putative RNA-binding protein 3	U	D		+	
2	RBMXL2	RNA-binding motif protein X-linked-like-2				+	
2	RCN3	Reticulocalbin-3				+	
2	RDX	Radixin				+	(200)
2	ROD1	Regulator of differentiation 1, PTBP3	U	D		+	(194)
2	RPF2	Ribosome production factor 2 homolog, BXDC1			+		
2	RPL11	60S ribosomal protein L11	U		+		
2	RPL12	60S ribosomal protein L12	U	D	+		(201)
2	RPL15	60S ribosomal protein L15		D	+		
3	RPL18	60S ribosomal protein L18		D	+		
2	RPL22	60S ribosomal protein L22		D		+	
16	RPL5	60S ribosomal protein L5		D	+		(202)
8	RPL6	60S ribosomal protein L6	U	D	+		(182)
8	RPL7	60S ribosomal protein L7	U	D	+		(203)
7	RPLP0	60S acidic ribosomal protein P0	U	D	+		(204)
4	RPLP2	60S acidic ribosomal protein P2	U	D	+		
3	RPS18	40S ribosomal protein S18	U	D	+		(205)
3	RPS19	40S ribosomal protein S19		D		+	(206)
3	RPS2	40S ribosomal protein S2	U	D	+		
4	RPS3	40S ribosomal protein S3	U	D		+	(207)
2	RPS3A	40S ribosomal protein S3a	U	D		+	
3	RPS4X	40S ribosomal protein S4, X isoform		D	+		
2	RPS8	40S ribosomal protein S8	U	D	+		
7	RPS9	40S ribosomal protein S9		D	+		(206)
13	RRBP1	Ribosome-binding protein 1	U	D		+	
2	SAE1	SUMO-activating enzyme subunit 1	U	D		+	(208)
4	SEPHS1	Selenide, water dikinase		D		+	(209)
2	SEPT2	Septin-2, NEDD5, DIFF6	U			+	(210)
3	SERPINE1	Plasminogen activator inhibitor 1	U	D		+	(211)
4	SERPINH1	Serpin H1, HSP47		D		+	(212)
6	SET	SET nuclear proto-oncogene	U	D	+		
6	SF3B1	Splicing factor 3B subunit 1	U	D	+		(213)
7	SF3B3	Splicing factor 3B subunit 3			+		(213)
3	SFPQ	Splicing factor, proline- and glutamine-rich	U	D		+	(214)
2	SFRS11	Splicing factor, arginine/serine-rich 11, SRSF11	U	D		+	
3	SFRS2	Splicing factor, arginine/serine-rich 2, SRSF2	U	D		+	(85)
2	SFRS7	Serine/arginine-rich splicing factor 7, SRSF7	U		+		(215)
3	SH3BGRL3	Putative uncharacterized protein, SH3 domain-binding glutamic acid-rich-like protein 3		D		+	
2	SKP1	S-phase kinase-associated protein 1	U	D		+	
2	SLC3A2	4F2 cell-surface antigen heavy chain, CD98	U	D	+		
4	SMS	Spermine synthase	U	D		+	
9	SND1	Staphylococcal nuclease domain-containing protein 1	U	D		+	
2	SNRNP200	U5 small nuclear ribonucleoprotein 200 kDa helicase		D	+		
3	SNRPA	U1 small nuclear ribonucleoprotein A	U			+	(216)
2	SNRPB	SnRNP-associated proteins B and B’	U	D	+		(217)
2	SNRPD1	Small nuclear ribonucleoprotein Sm D1	U		+		(218)
2	SNRPD2	Small nuclear ribonucleoprotein Sm D2		D	+		(219)
2	SNRPD3	Small nuclear ribonucleoprotein Sm D3		D	+		(218)
2	SNRPE	Small nuclear ribonucleoprotein E		D	+		(220)
37	SPTAN1	Highly similar to Spectrin alpha chain, brain	U	D		+	(221)
19	SPTBN1	Spectrin beta chain, brain	U	D	+		(222)
11	SSB	Lupus La protein	U			+	(21)
6	SSBP1	Single-stranded DNA-binding protein, mitochondrial			+		
4	SSRP1	FACT complex subunit SSRP1	U	D	+		(223)
3	ST13	Hsc70-interacting protein	U			+	(224)
2	STRBP	Spermatid perinuclear RNA-binding protein				+	
3	SUB1	Activated RNA polymerase II transcriptional coactivator p15	U	D		+	
2	SUMO1	Small ubiquitin-related modifier		D		+	(208)
4	SUPT16H	FACT complex subunit SPT16		D	+		
3	SYNCRIP	Heterogeneous nuclear ribonucleoprotein Q		D		+	
3	TFG	Trafficking from ER to Golgi regulator				+	
9	THBS1	Thrombospondin-1	U	D		+	(225)
29	TLN1	Talin-1	U	D		+	(226)
4	TLN2	Talin-2	U			+	
6	TNC	Tenascin C		D		+	(227)
3	TPD52L2	Tumor protein D54	U	D		+	
16	TPM1	Tropomyosin 1 alpha chain	U	D		+	(228)
17	TPM2	Tropomyosin beta chain	U	D		+	
6	TPM3	Tropomyosin alpha-3 chain	U	D		+	(229)
20	TPM4	Tropomyosin alpha-4 chain	U	D		+	(230)
2	TPP1	Tripeptidyl-peptidase 1	U	D	+		
4	TPR	Nucleoprotein TPR	U	D		+	(231)
4	TPT1	Tumor protein, translationally-controlled	U	D		+	
2	TROVE2	60 kDa SS-A/Ro ribonucleoprotein	U		+		
4	TUBA1C	Tubulin alpha-1C chain	U	D	+		(232)
6	TUBA4A	Tubulin alpha-4A chain, TUBA1	U	D	+		(233)
3	TUBB	Tubulin beta chain	U	D	+		(234)
2	TUBB1	Tubulin beta-1 chain			+		(233)
3	TUBB4B	Tubulin beta-2C, tubulin beta-4B, TUBB2C	U	D	+		(235)
2	TXN	Thioredoxin	U	D		+	(236)
2	TXNDC17	Thioredoxin domain-containing protein 17	U	D		+	
4	TXNDC5	Thioredoxin domain-containing protein 5	U	D		+	
2	TXNRD1	Thioredoxin reductase 1, cytoplasmic	U	D		+	(236)
8	UBA1	Ubiquitin-like modifier-activating enzyme 1	U			+	(237)
2	UCHL1	Ubiquitin carboxyl-terminal hydrolase isozyme L1	U	D		+	(238)
6	UGCGL1	UDP-glucose:glycoprotein glucosyltransferase 1		D		+	
18	UPF1	Regulator of nonsense transcripts 1		D	+		
3	USP5	Ubiquitin carboxyl-terminal hydrolase 5	U	D		+	
2	USP9X	Ubiquitin specific protease 9, X chromosome	U	D	+		
4	VASN	Vasorin	U	D		+	
4	VAT1	Synaptic vesicle membrane protein VAT-1 homolog	U	D		+	
3	VBP1	Von Hippel-Lindau binding protein		D		+	
13	VCL	Vinculin	U	D		+	(239)
15	VCP	Transitional endoplasmic reticulum ATPase	U	D		+	(240)
17	VIM	Vimentin	U	D	+		(241)
5	WARS	Tryptophanyl-tRNA synthetase, cytoplasmic	U	D		+	(242)
21	XRCC5	ATP-dependent DNA helicase 2 subunit 2, Ku80		D	+		(243)
21	XRCC6	ATP-dependent DNA helicase 2 subunit 1, Ku70	U	D	+		(244)
5	YBX3	D-binding protein A, CSDA, DBPA	U	D	+		(245)
5	YWHAB	14-3-3 protein beta/alpha	U	D		+	
9	YWHAE	14-3-3 protein epsilon	U	D		+	(246)
3	YWHAG	14-3-3 protein gamma	U	D		+	(246)
3	YWHAH	14-3-3 protein eta		D		+	(247)
5	YWHAQ	14-3-3 protein theta	U	D		+	(248)
5	YWHAZ	14-3-3 protein zeta/delta	U	D		+	(249)

# Pep., number of peptides identified by mass spectrometry; COVID (Up/Down), protein or gene expression up- and/or down-regulated in SARS-CoV-2 infected cells or patients; DS-affinity, concentration of NaCl (1.0 M, very high affinity, or 0.5 M, medium to high affinity) at which a DS-binding protein elutes from DS-affinity resin.

Of those not yet confirmed as autoAgs, a majority are similar to known autoAgs. As an example, we identified 18 ribosomal proteins, of which 9 have been individually identified as autoAgs (**Table 1**); however, anti-ribosomal autoantibodies are reported to react with a heterogeneous pool of many ribosomal proteins (206). Therefore, many of the ribosomal proteins we identified may be true but yet-to-be-confirmed autoAgs. As another example, autoantibodies against the 20S proteasome core are reported to be polyspecific and react with many subunits (250). Thus, although only 7 of 15 proteasome proteins we identified are thus far individually confirmed, the remainder may be true but yet-to-be-specified autoAgs. Similarly, some members of eukaryotic translation initiation and elongation factors are confirmed autoAgs, while others await confirmation. In summary, the putative autoantigen-ome from HFL1 cells provides at least 231 confirmed and 177 yet-to-confirm putative autoAgs (**Table 1**).

### DS-Affinity Proteins Are Functionally Connected and Enriched

**Figure 1:**
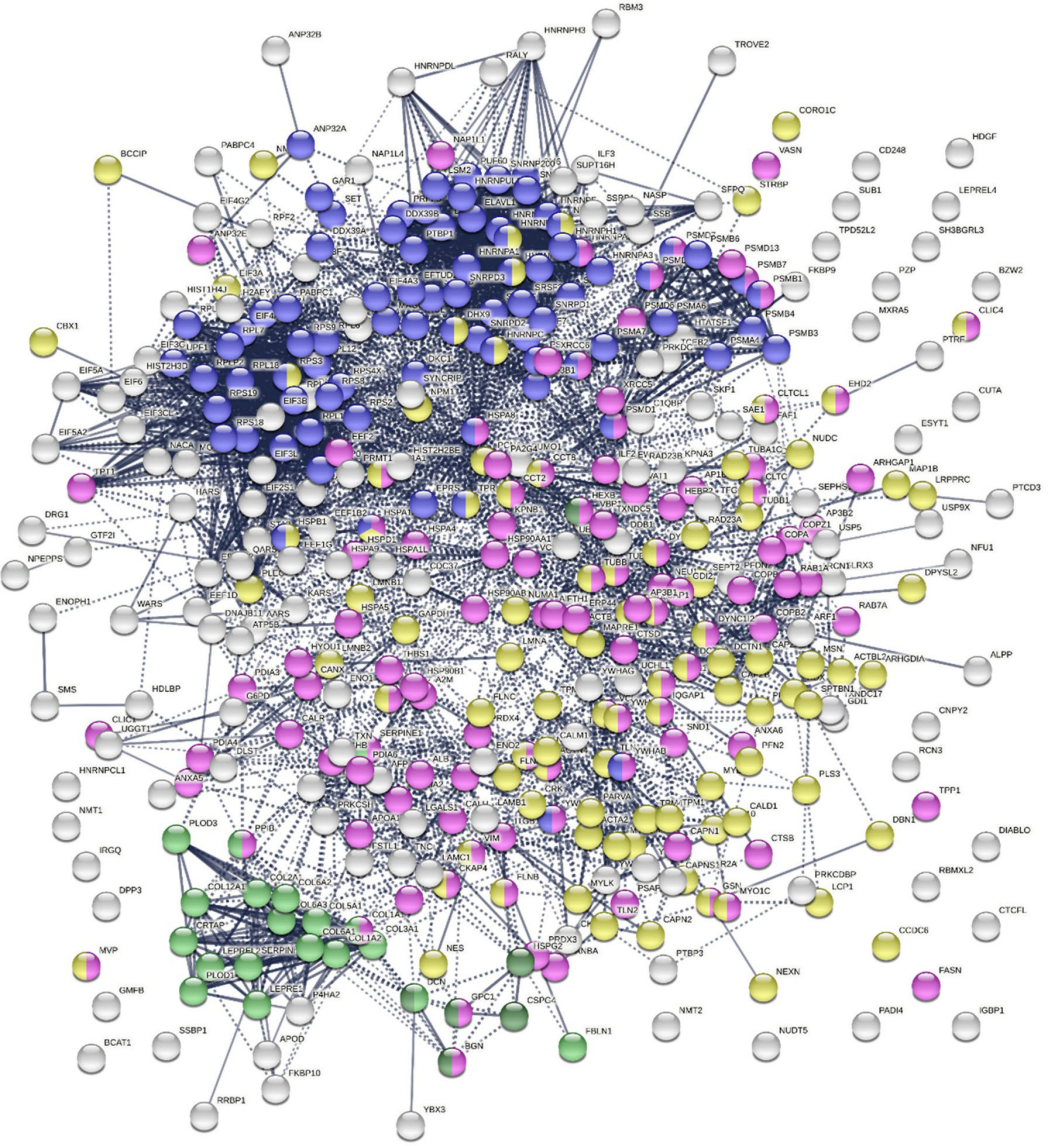
The 408-protein autoantigen-ome identified by DS-affinity from HFL1 cells forms a highly interacting network. Connecting lines represent interactions with high confidence (minimum interaction score of 0.7) as per STRING analysis. Colored proteins are involved in metabolism of RNA (blue), vesicles (pink), cytoskeleton (gold), collagen and elastic fibers (light green), and chondroitin sulfate/dermatan sulfate metabolism (dark green).

To find out whether DS-affinity-associated proteins are a random collection or biologically connected, we performed protein-protein interaction analyses with STRING (251). Of the 408 DS-associated proteins, 405 proteins recognized by STRING (ANP32C, ANXA2P2, HSP90AA2 excluded) have 7,582 interactions, whereas a random set of 405 proteins is expected to have only 3,060 interactions; hence, DS-affinity proteins represent a significantly connected network with PPI enrichment p-value <1.0E-6 (**Figure 1**). Based on cellular component classification, these proteins are highly concentrated in the nucleus (226 proteins), vesicles (111 proteins), ribonucleoprotein complexes (95 proteins), and the cytoskeleton (95 proteins).

Pathway and process analyses by STRING and Metascape (29) revealed that the mRNA metabolic process is the most enriched GO Biological Process, and the top KEGG pathways are the spliceosome and protein processing in the endoplasmic reticulum. The top Reactome pathways are metabolism of RNA, metabolism of proteins, and axon guidance. The top local network clusters are GTP hydrolysis and joining of the 60S ribosomal subunits and mRNA splicing. The Molecular Complex Detection algorithm identified clusters related to eukaryotic translation elongation, cellular responses to stress, regulation of RNA stability, COPI-independent Golgi-to-ER retrograde traffic, and supramolecular fiber organization.

### 352 Known and Putative AutoAgs Are COVID-Altered Proteins

To find out which autoAgs may be involved in COVID-19, we compared the DS-affinity autoantigen-ome with proteins and genes that are up- or down-regulated in SARS-CoV-2 infection (Coronascape database comparison, **Supplementary Table 1**) (29–49). Remarkably, 352 (86.3%) of the 408 DS-affinity proteins have been found to be altered (up- and/or down-regulated at protein and/or mRNA levels) in COVID-19 patients or SARS-CoV-2 infected cells (**Table 1**). Of these, 260 are reported as up-regulated and 303 as down-regulated (including 211 that are both up- and down-regulated). The numbers are not conflicting, because the COVID data were generated by multiple proteomic and transcriptomic methods and different cells and tissues. A protein may not be overexpressed even when its mRNA is up-regulated, and a protein/gene may be up-regulated in one tissue or patient but down-regulated in another tissue or patient. A protein is considered altered if it is up- or down-regulated at the protein or RNA level and, in relation to SARS-CoV-2 infection, it is considered a COVID-altered protein.

**Figure 2:**
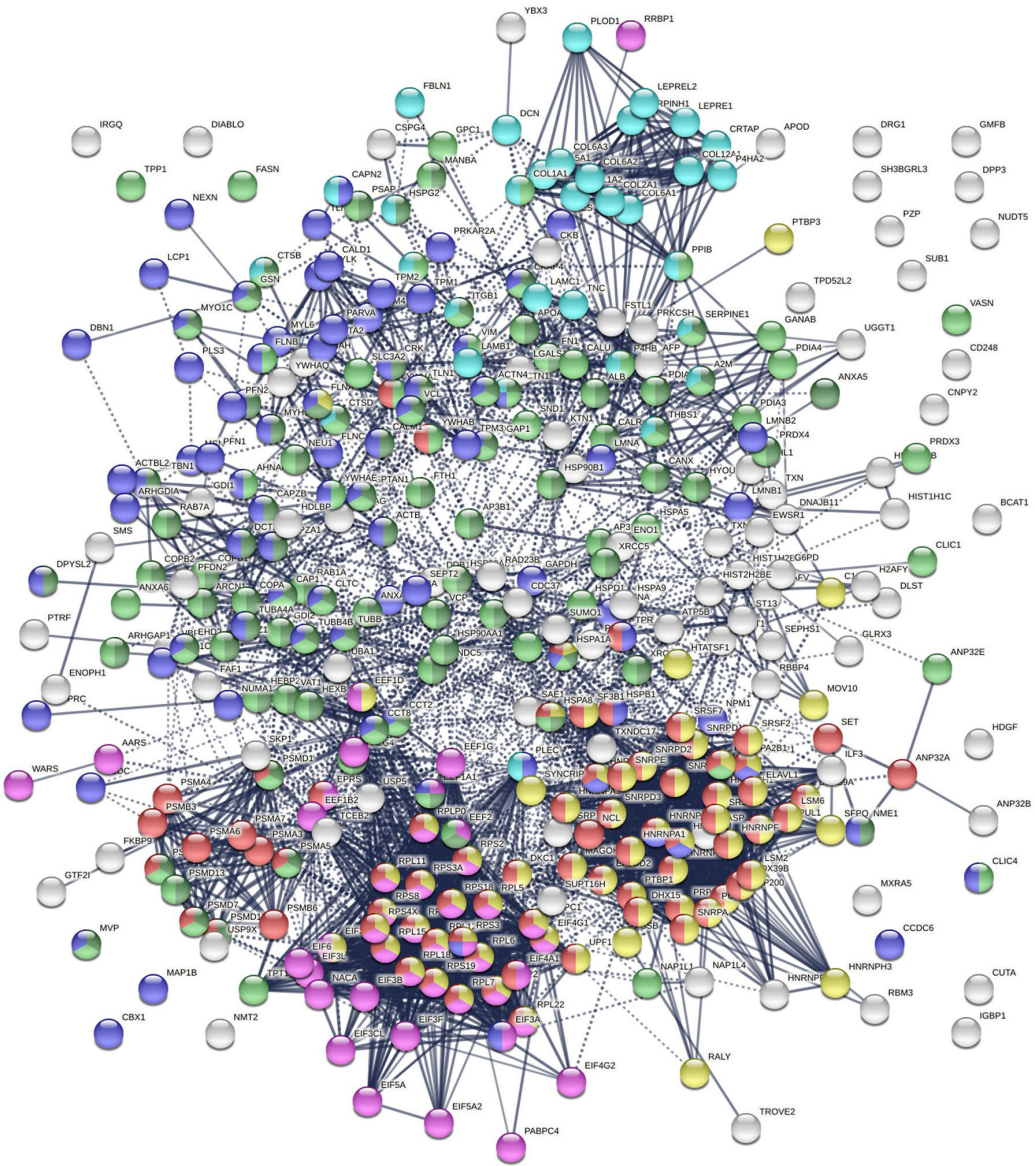
Network of 352 autoantigen-ome proteins that are altered in SARS-CoV-2 infected cells or patients. Connecting lines represent interactions with high confidence. Colored proteins are involved in metabolism of RNA (77 proteins, red), mRNA metabolic process (69 proteins, gold), translation (43 proteins, pink), vesicles (99 proteins, light green) and vesicle-mediated transport (84 proteins, dark green), cytoskeleton (84 proteins, blue), and extracellular matrix organization (32 proteins, aqua).

Protein-interaction analysis revealed that 352 COVID-altered proteins form a highly connected network, exhibiting 6,286 interactions (*vs*. 2,451 expected; PPI enrichment p-value <1.0E-6) (**Figure 2**). Based on cellular component analysis, the altered proteins can be located to intracellular organelles (323 proteins), nucleus (199 proteins), endomembrane system (143 proteins), vesicles (99 proteins), ribonucleoprotein complex (87 proteins), cytoskeleton (84 proteins), ER (72 proteins), and cell projections (52 proteins). Organelles with significant numbers of component proteins identified include the melanosome (30/105 proteins in melanosome), proteasome (16/64), polysome (13/66), spliceosome (34/187), ficolin-1-rich granule lumen (22/125), azurophil granules (17/155), and myelin sheath (26/157).

**Figure 3:**
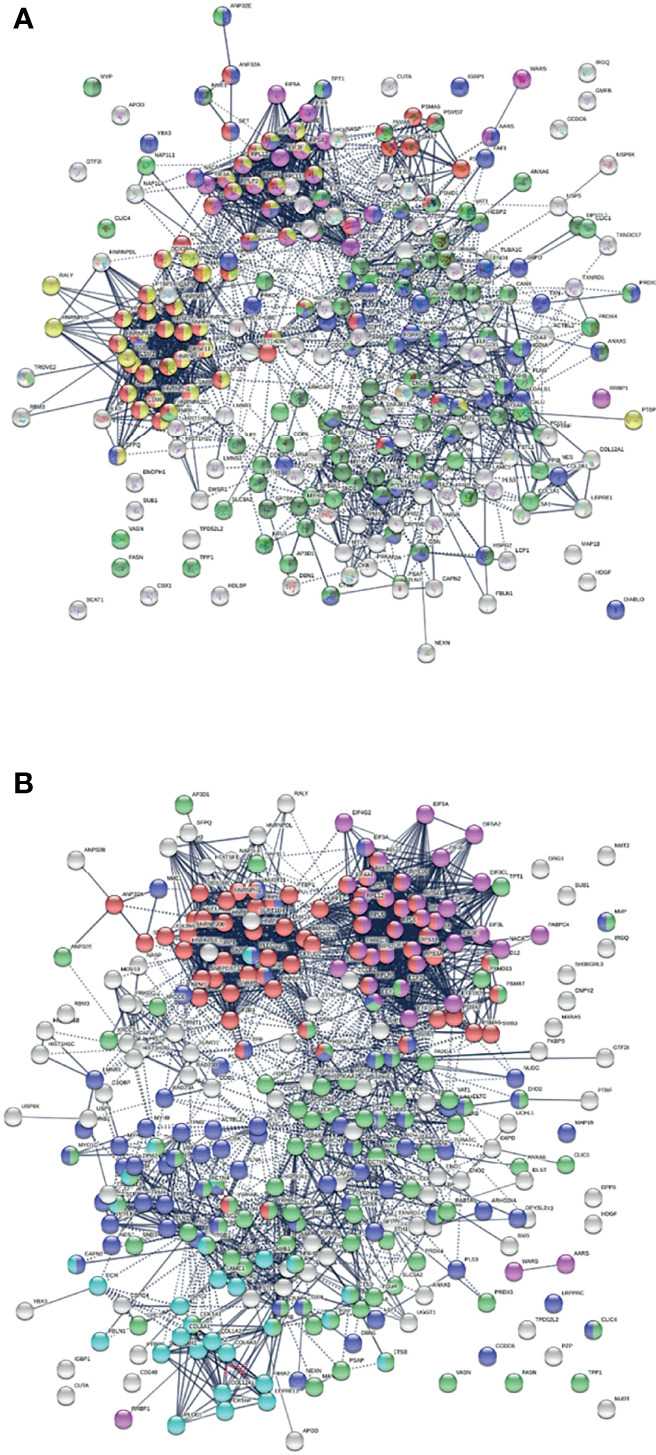
**(A)** Interaction network of 260 up-regulated proteins in SARS-CoV-2 infected cells or patients. Connecting lines represent interactions with high confidence (minimum interaction score of 0.7). Colored proteins are involved in metabolism of RNA (54 proteins, red), translation (28 proteins, pink), vesicles (82 proteins, light green) and vesicle-mediated transport (67 proteins, dark green), regulation of cell death (61 proteins, blue), and mRNA metabolic process (46 proteins, gold). **(B)** Interaction network of 303 down-regulated proteins in SARS-Cov-2 infected cells and patients. Connecting lines represent interactions with high confidence. Marked proteins are involved in RNA metabolism (64 proteins), translation (39 proteins, pink), vesicles (88 proteins, green), cytoskeleton (73 proteins, blue), and extracellular matrix organization (29 proteins, aqua).

Similarly, the group of 260 up-regulated proteins is highly connected (3,747 interactions *vs*. 1,424 expected) with significant enrichment in proteins associated with RNA and mRNA metabolism, translation, vesicles and vesicle-mediated transport, and regulation of cell death (**Figure 3A**). The group of 303 down-regulated proteins is also highly connected (4,860 interactions *vs*. 1,907 expected), and these proteins are significantly related to RNA metabolism, translation, vesicles, cytoskeleton, and extracellular matrix (**Figure 3B**).

### Pathways and Processes Affected by COVID-Altered Proteins

Network enrichment analysis by Metascape revealed that the 352 COVID-altered proteins are most significantly enriched in RNA metabolism, axon guidance, and translation (**Table 2**). Many processes, e.g., regulated exocytosis, wound healing, supramolecular fiber organization, smooth muscle contraction, and platelet degranulation are significantly affected by COVID-altered proteins regardless of whether they are up- or down-regulated. The up-regulated proteins are more related to axon guidance and interleukin signaling, whereas down-regulated proteins are more related to cellular response to stress and apoptosis.

**Table 2:** Top enriched pathways and processes related to COVID-altered proteins.

COVID	Ontology	Description	Count	%	Log_10_(P)
**Altered**	R-HSA-8953854	Metabolism of RNA	78	22.16	-51.2
	R-HSA-422475	Axon guidance	63	17.90	-40.6
	GO:0006412	Translation	66	18.75	-35.9
	GO:0000377	RNA splicing	44	12.50	-28.0
	GO:0045055	Regulated exocytosis	58	16.48	-26.7
	GO:0006457	Protein folding	33	9.38	-24.3
	R-HSA-1474244	Extracellular matrix organization	33	9.38	-20.6
	GO:0043687	Post-translational protein modification	35	9.94	-20.0
	GO:0071826	Ribonucleoprotein complex subunit organization	32	9.09	-19.7
	CORUM:5615	Emerin complex 52	13	3.69	-18.8
	GO:0010638	Positive regulation of organelle organization	40	11.36	-16.1
	GO:0042060	Wound healing	38	10.80	-15.6
	GO:0006913	Nucleocytoplasmic transport	30	8.52	-15.6
	R-HSA-114608	Platelet degranulation	19	6.27	-15.6
	R-HSA-5653656	Vesicle-mediated transport	40	11.36	-15.4
	GO:0097435	Supramolecular fiber organization	41	11.65	-15.1
	CORUM:1335	SNW1 complex	10	3.30	-15.1
	GO:0002181	Cytoplasmic translation	18	5.11	-15.1
	R-HSA-445355	Smooth muscle contraction	13	3.69	-14.9
	GO:0031647	Regulation of protein stability	27	7.67	-14.9
**Up**	R-HSA-72163	mRNA splicing - major pathway	23	8.85	-18.8
	R-HSA-449147	Signaling by interleukins	26	10.00	-12.6
	GO:0000904	Cell morphogenesis involved in differentiation	31	11.92	-11.3
**Down**	R-HSA-2262752	Cellular responses to stress	55	18.15	-34.5
	R-HSA-109581	Apoptosis	23	7.59	-17.5
	GO:0035966	Response to topologically incorrect protein	22	7.26	-15.0

Count, number of DS-affinity proteins with membership in the given ontology term. %, percentage of DS-affinity proteins in the given ontology term.

### COVID-Altered AutoAgs Are Strongly Related to the Nervous System

**Figure 4:**
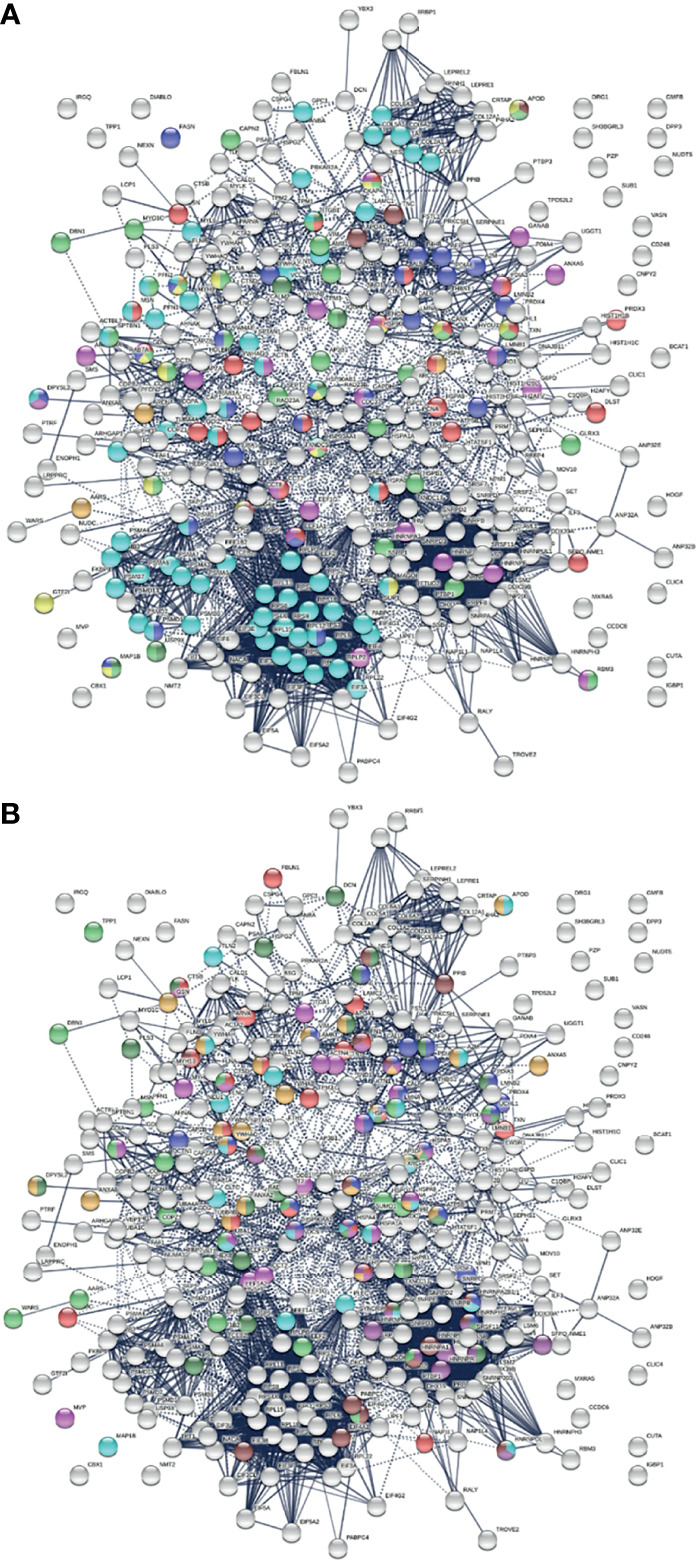
**(A)** Nervous system-related proteins among COVID-altered proteins. Colored proteins are involved in axon guidance (62 proteins, aqua), axon growth cone (25 proteins, blue), myelin sheath (26 proteins, red), neuron projection (32 proteins, green) and neuron projection extension (7 proteins, dark green), neuronal cell body (16 proteins, gold), peripheral nervous system axon regeneration (3 proteins, brown), cerebellar Purkinje cell layer development (4 proteins, amber), and olfactory bulb (23 proteins, pink). **(B)** Neurological disease-related proteins among proteins altered in COVID. Colored are proteins found in neuronal infection with Japanese encephalitis virus (23 proteins, blue), neuroblastoma (21 proteins, red), glioblastoma (22 proteins, pink), neurodegeneration in Down syndrome (26 proteins, dark green), Alzheimer disease (22 proteins, aqua), schizophrenia (24 proteins, amber), cerebral ischemia induced neurodegenerative diseases (17 proteins, dark purple), Parkinson disease (17 proteins, brown), and neurodegeneration (21 proteins, green).

COVID-19 patients frequently report neurological problems, such as loss of smell and taste, dizziness, headache, and stroke. While most symptoms are transient, some recovered patients are haunted by lingering neurological and psychological problems long after the viral infection. The underlying cause of transient and long-lasting neurological effects of COVID-19 has been puzzling. Analysis of COVID-altered proteins revealed a strong link to the nervous system. Of the 352 COVID-altered proteins, at least 150 are related to the nervous system (**Figure 4A**). More than 60 proteins are related to axon guidance based on ontology analyses (**Table 2** and **Figure 4A**). In addition, there are 39 proteins related to neuron projection, 26 proteins related to myelin sheath, 25 proteins related to axon growth cone (252), 16 proteins related to neuronal cell body, 4 proteins related to cerebellar Purkinje cell layer, 3 proteins related to peripheral nervous system axon regeneration, and 2 proteins related to radial glial scaffolds. In particular, we found that 23 COVID-altered proteins are related to the olfactory bulb (253), which may explain the loss of smell in many COVID-19 patients.

Most of these proteins are known autoAgs, e.g., ACTB, CANX, A2M, APOA1, CAPZA1, DPYSL2, FLNA, GDI2, LGALS1, MSN, PDIA3, PFN2, TNC, UCHL1, VCP, and VCL (see autoAg references in **Table 1**). Some yet-to-be-confirmed autoAgs with direct relation to the nerve system, e.g., NES (expressed mostly in nerve cells) and APOD (expressed by oligodendrocytes), warrant further investigation.

The COVID-altered proteins are also associated with a number of neurological diseases (**Figure 4B**). By comparing our data with published proteomes, 23 proteins were similarly found in neuronal infection by Japanese encephalitis virus (254), 21 proteins in neuroblastoma (255), 22 proteins in glioblastoma (256), 26 proteins in neurodegeneration in Down syndrome (257), 22 proteins in Alzheimer disease hippocampus (258), 24 proteins in schizophrenia (259), 17 proteins in cerebral ischemia (260), and 17 proteins in Parkinson disease (261).

Coronavirus-induced demyelination has been reported in a mouse model of multiple sclerosis (262), which may explain our identification of 26 altered proteins related to the myelin sheath in SARS-CoV-2 infection. In a mouse brain injury model, DS appears to play an important role in glial scar formation and regeneration of dopaminergic axons (263). Alterations of white matter DS and extracellular matrix are specific, dynamic, and widespread in multiple sclerosis patients (264). DS has recently been reported to promote neuronal differentiation in mouse and human neuronal stem cells (265). Given the various functional roles of DS, our identification of a large number of known and putative autoAgs with DS affinity related to the nervous system is a compelling finding.

### COVID-Altered AutoAgs Are Related to Cell Death, Wound Healing, and Blood Coagulation

**Figure 5:**
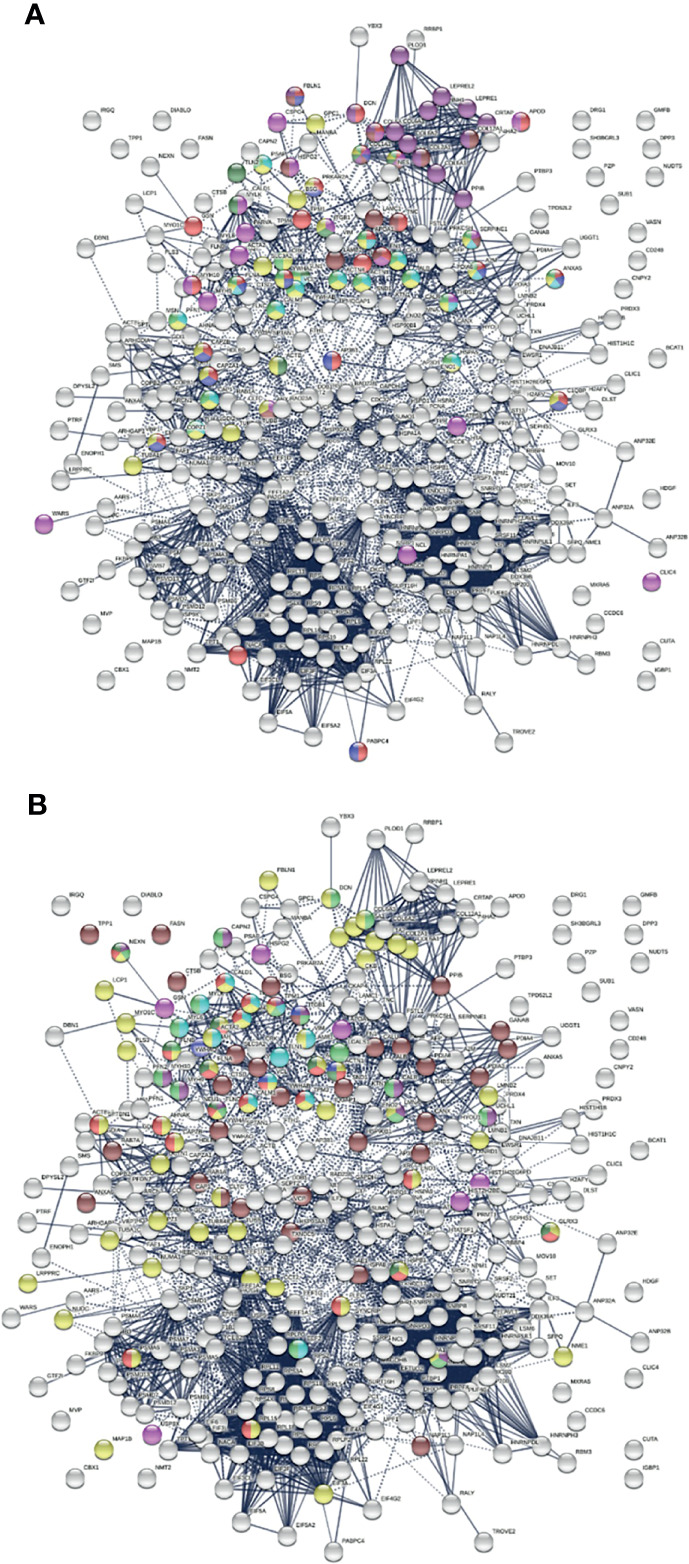
**(A)** Relation of COVID-altered proteins to wound healing and hemostasis. Response to wounding (25 proteins, red), blood vessel development (20 proteins, pink), blood coagulation (14 proteins, blue), collagen-containing extracellular matrix (13 proteins, brown), collagen biosynthesis and modifying enzymes (16 proteins, dark purple), platelet activation (3 proteins, dark green) and platelet activation signaling and aggregation (22 proteins, green), platelet degranulation (18 proteins, aqua), and hemostasis (35 proteins, gold). **(B)** Other significantly enriched groups among altered proteins. Supramolecular fiber (56 proteins, amber), melanosome (30 proteins, brown), striated muscle cell differentiation (11 proteins, purple), myofibril (23 proteins, red), muscle structure development (18 proteins, green), muscle contraction (13 proteins, aqua), Z disk (9 proteins, dark green), intercalated disk (4 proteins, blue), and amyloid fiber formation (6 proteins, pink).

SARS-CoV-2 infection causes host cell death and leads to tissue injury. Wound healing, cellular response to stress, and apoptosis are among the most significant processes related to COVID-altered proteins (**Table 2** and **Figure 5A**). For example, we identified 66 proteins related to regulation of cell death and 23 related to regulation of apoptotic signaling pathways. DS binds to apoptotic cells and autoAgs released from dying cells, which has led to our previous identification of hundreds of autoAgs (13–16, 18). Upon tissue injury, DS biosynthesis is ramped up by fibroblasts and epithelial and endothelial cells (7–9). After tissue injury, DS assists fibroblast migration into the wound to facilitate granulation tissue formation and wound healing (11). DS, similar to heparin, is also an important anticoagulant that inhibits clot formation *via* interaction with antithrombin and heparin cofactor II (266). Given these biological roles of DS, it is consistent that a large number of COVID-altered proteins related to cell death and tissue injury are identified by DS-affinity.

Blood coagulation and thrombosis are frequent complications of COVID-19. Platelet degranulation is found to be significantly associated with at least 18 altered proteins (**Table 2** and **Figure 5A**). COVID-altered proteins are related to blood coagulation, platelet activation, platelet alpha granules, fibrinogen binding, fibrinogen complex, platelet plug formation, von Willebrand factor A-like domain superfamily, and platelet-derived growth factor binding. Collagens, which support platelet adhesion and activation, and collagen biosynthesis and modifying enzymes are also among the COVID-altered proteins, e.g., collagen type VI trimer and type I trimer (**Figure 5A**). The majority of these altered proteins are known autoAgs, e.g., ALB, ANXA5, C1QBP, CALM1, CAPZB, COL1A1, COL1A2, COL6A1, FBLN1, FN1, PLEC, PPIB, THBS1, TLN1, TUBA4A, and YWHAZ (see autoAg references in **Table 1**). Some are unknown and await further investigation, e.g., AP3B1, CRK, CTSB, EHD2, PLOD1, PSAP, and PARKAR2A.

### Supramolecular Fibril Alteration Offers Clues to Muscle Dysfunction and Fibrosis

Over 50 supramolecular filament proteins are identified by DS-affinity from HFL1 cells. Remarkably, nearly all (except for one) are found to be altered in SARS-CoV-2 infection, and the majority have already been reported as autoAgs (**Table 1**). They include various isoforms of actin, actinin, collagen, filamin, fibronectin, fibulin, dynactin, dynein, lamin, myosin, nestin, nexilin, profilin, plectin, plastin, proteoglycan, septin, spectrin, talin, tropomyosin, tubulin, vinculin, and vimentin (**Table 1** and **Figure 5B**). These proteins are major components of the extracellular matrix, basement membrane, cell cytoskeleton, cytoskeletal motors, muscle filaments, and contractile motors of muscle cells.

A significant number of COVID-altered proteins are related. Emerin complex and smooth muscle contraction are among the top enriched biological processes of COVID-altered proteins (**Table 2** and **Figure 5B**). Emerin is highly expressed in cardiac and skeletal muscle, and emerin mutations cause X-linked recessive Emery-Dreifuss muscular dystrophy, cardiac conduction abnormalities, and dilated cardiomyopathy. Smooth muscle resides primarily in the walls of hollow organs where it performs involuntary movements, e.g., respiratory tract, blood vessels, gastrointestinal tract, and renal glomeruli. In addition, we identified proteins with significant association to myofibrils (the contractile elements of skeletal and cardiac muscle; 23 proteins) (**Figure 5B**), stress fiber (a contractile actin filament bundle that consists of short actin filaments with alternating polarity: MYH9, MYLK, FLNB, TPM1, TPM2, TPM3, TPM4, ACTN1, ACTN4), muscle filament sliding (the sliding of actin thick filaments and myosin thick filaments past each other in muscle contraction), Z disk (plate-like region of a muscle sarcomere to which the plus ends of actin filaments are attached), intercalated disc (a cell-cell junction complex at which myofibrils terminate in cardiomyocytes, mediates mechanical and electrochemical integration between individual cardiomyocytes), and negative regulation of smooth muscle cell-matrix adhesion (2 proteins; SERPINE1, APOD).

Pulmonary fibrosis is prominent in COVID-19 and contributes to lethality in some cases (267, 268). Fibrosis, or fibrotic scarring, is pathological wound healing in which excessive extracellular matrix components are produced by fibroblasts and accumulate in the wounded area. Histopathological examination of COVID-19 patients found highly heterogenous injury patterns reminiscent of exacerbation of interstitial lung disease, including interstitial thickening, fibroblast activation, and deposition of collagen fibrils (22). We identified a significant number of COVID-altered proteins that are associated with collagen bundles and collagen biosynthesis and modifying enzymes (16 proteins), extracellular matrix organization (33 proteins), supramolecular fibers, and amyloid formation offering functional links to fibrosis (**Figure 5B**).

### Potential AutoAgs in COVID-19 Patients and a Connection to the Melanosome

**Figure 6:**
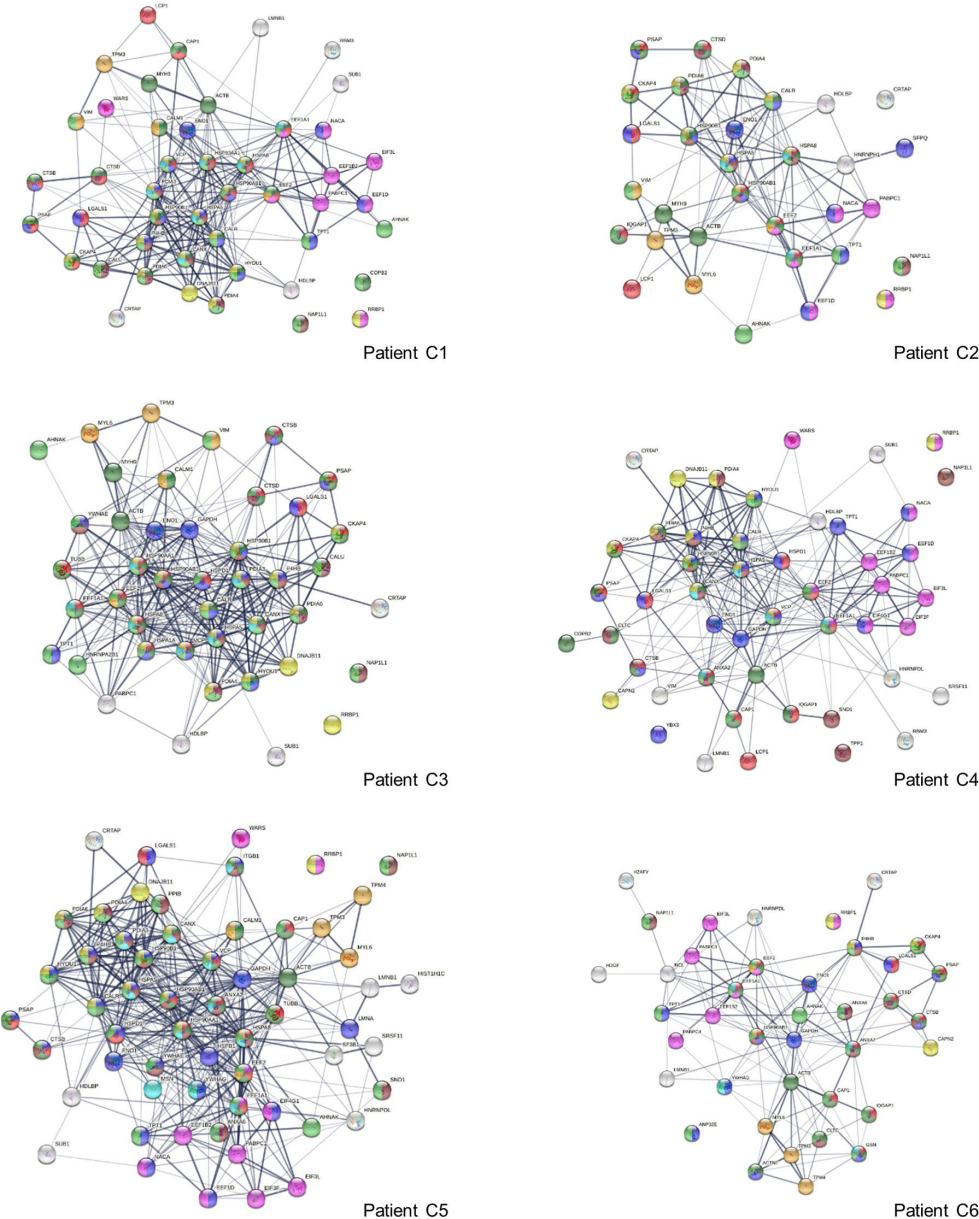
Interaction network of altered proteins in 6 COVID-19 patients. Colored proteins are associated with leukocyte activation involved in immune response (red), vesicles (light green) and vesicle-mediated transport (dark green), protein processing in the ER (yellow), regulation of cell death (blue), translation (pink), melanosome (brown), myelin sheath (aqua), and muscle contraction (amber).

To find out how altered proteins may differ in patients, we compared our putative autoantigen-ome to published single-cell RNA sequencing data of 6 patients hospitalized for COVID-19 (29, 35) and identified 32-59 putative autoAgs per patient (**Figure 6**). Interestingly, while identified from different patients, the altered proteins/genes identified share involvement of leukocyte activation, vesicles and vesicle transport, protein processing in the ER (including antigen processing and presentation), regulation of cell death, translation, muscle contraction, myelin sheath, and curiously, the melanosome (**Figure 6**). The estrogen signaling pathway and thyroid hormone synthesis are found to be associated with altered proteins in some patients. Patient C2 has 5 altered proteins related to neuron differentiation regulation, and patient C4 has 6 altered proteins related to neuron death.

Eleven altered proteins were identified in all 6 patients, including known autoAgs (ACTB, EEF1A1, EEF2, ENO1, LGALS1, PABPC1) and unknown ones (CRTAP, NAP1L1, PSAP, RRBP1, TPT1) (**Table 1**). AHNAK (neuroblast differentiation-associated protein, a known autoAg in lupus) was identified in 5 patients. Overall, a majority of the altered proteins identified from the 6 COVID patients are known autoAgs, e.g., CALM1, CALR, CALU, CANX, DNAJB11, HDGF, HSPA5 (BiP), IQGAP1, LCP1, LMNB1, MYH9, NACA, P4HB, SFPQ, PDIA3, TPM3, TUBB, VCP, VIM, WARS, and YB3 (**Table 1**). Unknown or putative autoAgs include CAP1, CTSB, HDLBP, HYOU1, SND1, and SUB1.

We initially identified 30 DS-affinity proteins from HFL1 cells related to the melanosome, and, intriguingly, all of these are also COVID-altered proteins (**Figure 5B**). Based on STRING GO analysis, the melanosome is the most significant cellular component related to altered proteins in all 6 patients (with false discovery rates ranging from 1.52E-8 to 1.11E-23). In HIV infection, melanosome production is stimulated in some patients and leads to an increase in pigmented lesions (269). However, melanosome involvement in COVID-19 is not known. Two Wuhan doctors in intensive care for COVID temporally turned dark, although the cause was thought to be a drug reaction. A COVID patient has been reported with acute flaccid tetraparesis and maculopapular pigmented plaques on the limbs (270). In mice, coronavirus induces an acute and long-lasting retinal disease, with initial retinal vasculitis followed by retinal degeneration that is associated with retinal autoantibodies and retinal pigment epithelium autoantibodies (271). Future research will be needed to investigate the interaction between COVID and melanosome activation.

### Association Between Autoimmunity and Virus Infections

**Figure 7:**
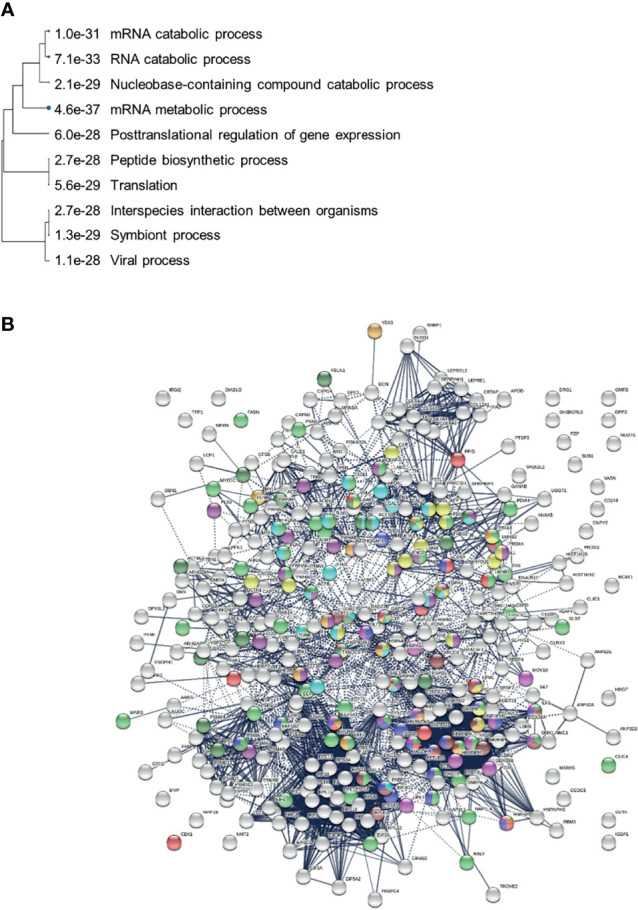
**(A)** Hierarchical clustering of top 10 pathways involving COVID-altered proteins. Analysis based on hypergeometric distribution followed by FDR correction. **(B)** COVID-altered host proteins with DS-affinity found in various viral infections. Porcine reproductive and respiratory syndrome (56 proteins, green), H5N1 avian influenza virus (27 proteins, dark purple), Japanese encephalitis virus (23 proteins, gold), Rift Valley fever virus (24 proteins, aqua), Hepatitis B virus (22 proteins, dark green), HIV (identified in different studies, 18 amber, 18 brown, 18 red and 17 pink), and shared among positive-sense RNA viruses (20 proteins, blue).

We identified COVID-altered proteins with DS-affinity that are involved in the host response to various aspects of viral infection and that possess a high propensity to become autoAgs. For example, viral RNA metabolism, translation, vesicles, and vesicle transport contribute a large number of known and putative autoAgs. In addition, viral processes, particularly symbiont processes and interspecies interactions between host and viruses, contribute significantly to altered proteins (**Figure 7A**). For example, among altered proteins related to response to viral processes, HSPA8, DDB1, RAD23A, PABPC1, PPIB, P4HB, LGALS1, GSN, and ILF3 are known autoAgs (**Table 1**).

In particular, COVID-altered cytoskeletal filament proteins shed light on viral trafficking in host cells. SARS-CoV-2 infection induces profound remodeling of the cytoskeleton, and replicating viral vesicles are surrounded by a network of intermediate filaments (272). The cytoskeletal network appears to facilitate coronavirus transport and expulsion, with thickening actin filaments providing the bending force to extrude viral vesicles (273). We identified 84 altered proteins related to the cytoskeleton and 84 altered proteins related to vesicle-mediated transport (**Figure 2**). These altered proteins are implicated in various processes, including cytoskeleton-dependent intracellular transport, actin fiber-based movement, actin-mediated cell contraction, microtubule-dependent trafficking from the Golgi to the plasma membrane, and transport along microtubules.

Many positive-strand RNA viruses (including SARS-CoV-2, Enterovirus, Hepatitis C virus, Norovirus, and Poliovirus) hijack a common group of nuclear factors to support the biosynthetic functions required for viral replication and propagation (274). 20 of these hijacked nuclear proteins are identified by DS-affinity in our study (**Figure 7**). In addition, altered proteins are found in other viral infections, including porcine reproductive and respiratory syndrome virus (275), H5N1 avian influenza viruses (276, 277), Japanese encephalitis virus (254), Rift Valley fever virus (278), Hepatitis B virus (279), HIV (280–282), Herpes Simplex virus (283), and Epstein-Barr virus infection (**Figure 7B** and STRING ontology analysis). In some cases, viral infections may have both enhancing and protective effects on autoimmunity in type 1 diabetes (284).

Our study identified a large number of known and putative autoAgs that are related to mRNA metabolism, translation, vesicles, and vesicle trafficking (**Figures 1**, **2**). This finding begs us to wonder whether mRNA vaccines may induce unintended autoimmune consequences in the long term. To induce protective immunity, mRNA vaccine vesicles will need to be transported into cells where they use the host cell machinery to produce a viral protein antigen, whereupon the antigen will be processed and presented by MHC molecules to induce B and T cell responses.

mRNA translation requires ribosomes, translation initiation factors, aminoacyl-tRNA synthetases, and elongation factors. We identified 18 ribosomal proteins by DS-affinity, all of which are altered in SARS-CoV-2 infection and 9 of which are known autoAgs (see references in **Table 1**). We also identified 15 eukaryotic translation initiation factor proteins, with 12 of them being COVID-altered and 4 being known autoAgs (**Table 1**). Six elongation factor proteins (5 subunits of EEF1 complex, EEF2) were identified by DS-affinity, of which all 6 are COVID-altered and 3 are known autoAgs (**Table 1**). Six tRNA synthetases were identified, with 5 being known autoAgs and 3 (AARS, EPRS, WARS) COVID-altered (**Table 1**). Autoantibodies to AARS are associated with interstitial lung disease and myositis (51, 285). EPRS appears to regulate pro-fibrotic protein synthesis during cardiac fibrosis (286). Gene mutations of WARS cause an autosomal dominant neurologic disorder characterized by slowly progressive distal muscle weakness and atrophy affecting both the lower and upper limbs (242, 287).

Once synthesized, the exogenous protein antigens are degraded by proteasomes, and the resulting peptides are transported into the ER where they are loaded onto MHC molecules by peptide loading complexes for presentation to T cells. In relation to these steps, 15 proteasome subunits were identified by DS-affinity, with 12 being COVID-altered and 7 being known autoAgs (**Table 1**). Nine proteins related to antigen processing and presentation are found to be altered in the 6 COVID-19 patients analyzed in this study, including HSPA1A, HSPA8, HSP90AA1, HSPAB1, HSPA5, PDIA3, CANX, CALR, and CTSB, with 7 being known autoAgs (**Figure 5** and **Table 1**).

In addition, among the 352 COVID-altered proteins identified in this study, 69 proteins are associated with mRNA metabolism (**Figure 2**). Many of these proteins may be irrelevant to non-replicating mRNA molecules in mRNA vaccines, however, some are likely needed in processes such as 3’ end processing, deadenylation, and nonsense-mediated decay. For example, we identified poly(A) tail binding proteins PABPC1 and PABPC4 as COVID-altered proteins, both of which have been reported as autoAgs (**Table 1**).

Our study identified 99 altered proteins associated with vesicles and 84 proteins associated with vesicle-mediated transport (**Figures 1**, **2**, **5**). Although it is not clear which host molecules are involved in extra- and intracellular transport and uptake of mRNA vaccine vesicles, some of the vesicle-related proteins identified as DS-affinity proteins may be involved, e.g., proteins of receptor-mediated endocytosis (APOA1, CALR, CANX, CAP1, CLTC, HSP90AA1, HSP90B1, HSPG2, ITGB1, YWHAH) or phagocytosis (ACTB, CRK, GSN, HSP90AA1, HSP90AB1, MYH9, MYO1C, PDIA6, RAB7A, THBS1, TXNDC5).

Overall, a significant number of autoAgs related to different steps of mRNA vaccine action were identified in this study; however, our findings do not mean that these autoAgs will lead to aberrant autoimmune reactions as a result of mRNA vaccination. The development of autoimmune diseases or autoimmunity-related diseases entails a complex cascade of molecular and cellular interactions. Long-term monitoring of autoimmune adverse effects will be needed.

## Conclusion

This study identifies an autoantigen-ome of 408 proteins from human fetal lung fibroblast HFL1 cells by DS-affinity and protein sequencing, of which at least 231 proteins are confirmed autoAgs. Of these, 352 (86.3%) are found to be altered in SARS-CoV-2 infection when compared to published data, with at least 210 COVID-altered proteins being known autoAgs. The altered proteins are significantly enriched in a number of pathways and processes and are closely connected to various disease manifestations of COVID-19, particularly neurological problems, fibrosis, muscle dysfunction, and thrombosis.

Viral infections cause significant perturbations of normal cellular and tissue component molecules in the host, leading to cell death and tissue injury. Autoantigens resulting from molecular alterations may result directly from the injury or indirectly from responses to the injury. As a stress response, DS biosynthesis may be ramped up to facilitate wound healing and dead cell clearance. DS associates with autoAgs and stimulates autoreactive B cells and autoantibody production. Specific autoantibodies that are initially induced in response to a certain injury site may circulate and attack secondary sites where the autoAgs are also expressed, leading to a complex array of local and systemic autoimmune diseases.

This study supports a connection between COVID and autoimmunity. We have shown in a series of papers on autoimmune disease that proteins with high affinity for DS possess intrinsic propensity to become recognized by the humoral immune system and serve as autoantigens (12–16, 18). We have shown in a prior paper that proteins that are, by themselves, not immunogenic can be turned into potent autoantigens and induce an autoantibody response if they are engineered to bind to DS and are exposed as DS-autoAg complexes to the immune system (14). The list of proteins enriched by DS-affinity in lung fibroblasts is, at first, only a putative catalogue of autoantigens. Intriguingly, when we performed a literature analysis of all DS-enriched proteins, we found that a very high proportion of them correspond to known autoantigens (this enrichment is much higher than would be expected by chance). Many of the COVID-induced autoantibodies described in a recent study correspond to autoantigens identified in our study (e.g., ribosomal P proteins, Ro/La, U1-snRNP, and chromatin histones) (288). While likely also autoantigens, we label proteins that have not been observed as autoantigens in the literature as “putative autoAgs.” We then show that among the DS-affinity proteins, there are many proteins that are also affected by COVID (many more than would be expected by statistical chance). Taking all these observations together, we hypothesize that our findings provide a rationale for why SARS-CoV-2 infection may induce autoimmune sequelae. Future serological studies will be needed to further confirm this hypothesis, but our dataset, together with the comprehensive list of possible autoAg targets, will be a valuable guide and map for these ongoing investigations. We believe that our dataset will be of great interest and value for research groups worldwide that are attempting to tackle the autoimmune aspects of COVID.

The COVID-19 autoantigen-ome provides a detailed molecular map for investigating the diverse spectrum of autoimmune sequelae caused by the pandemic. The COVID autoantigen atlas we are establishing will serve as a detailed molecular map and reference for ongoing research into COVID-induced autoimmunity and possible autoimmune causes of “long COVID” syndrome. It will thus serve as an important resource for the scientific community.

## Materials and Methods

### HFL1 Cell Culture

The HFL1 cell line was obtained from the ATCC (Manassas, VA, USA) and cultured in Eagle’s Minimum Essential Medium supplemented with 10% fetal bovine serum (Thermo Fisher) and a penicillin-streptomycin-glutamine mixture (Thermo Fisher) at 37°C.

### Protein Extraction

About 100 million cells were harvested and suspended in 10 ml of 50 mM phosphate buffer (pH 7.4) containing the Roche Complete Mini protease inhibitor cocktail. Cells were homogenized on ice with a microprobe sonicator until the turbid mixture became nearly clear with no visible cells left. The homogenate was centrifuged at 10,000 g at 4°C for 20 min, and the supernatant was collected as the total protein extract. Protein concentration was measured with the RC DC protein assay (Bio-Rad).

### DS-Sepharose Resin Preparation

20 ml of EAH Sepharose 4B resins (GE Healthcare Life Sciences) were washed with distilled water three times and mixed with 100 mg of DS (Sigma-Aldrich) in 10 ml of 0.1 M MES buffer, pH 5.0. 500 mg of N-(3-dimethylaminopropyl)-N’-ethylcarbodiimide hydrochloride (Sigma-Aldrich) powder was added to the mixture. The reaction proceeded by end-over-end rotation at 25°C for 16 h. After coupling, resins were washed with water and equilibrated first with a low-pH buffer (0.1 M acetate, 0.5 M NaCl, pH 5.0) and then with a high-pH buffer (0.1 M Tris, 0.5 M NaCl, pH 8.0).

### DS-Affinity Fractionation

The total proteins extracted from HFL1 cells were fractionated on DS-Sepharose columns with a BioLogic Duo-Flow system (Bio-Rad). About 40 mg of proteins in 40 ml of 10 mM phosphate buffer (pH 7.4; buffer A) were loaded onto the column at a rate of 1 ml/min. Unbound proteins were washed off with 60 ml of buffer A, and weakly bound proteins were eluted with 40 ml of 0.2 M NaCl in buffer A. DS-binding proteins were eluted with sequential 40-ml step gradients of 0.5 M and 1.0 M NaCl in buffer A. Fractions were desalted and concentrated to 0.5 ml with 5-kDa cut-off Vivaspin centrifugal filters (Sartorius). Fractionated proteins were separated by 1-D SDS-PAGE in 4-12% Bis-Tris gels, and the gel lanes corresponding to 1.0 M or 0.5 M NaCl elutions were divided into two or three sections for sequencing.

### Mass Spectrometry Sequencing

Fractionated proteins with different affinity to DS were separated on 1D SDS PAGE in 4-12% NuPAGE Novex Bis-Tris gels (Invitrogen). Based on protein band intensity, the protein lanes containing proteins eluting at 0.5 M or 1.0 M NaCl were each cut into 2 sections, containing top and bottom bands, respectively. Gel sections were transferred into 1-mL tubes, cut into 1-mm^3^ pieces, dehydrated with acetonitrile, and dried in a speed-vac. Protein sequencing was performed at the Taplin Biological Mass Spectrometry Facility at Harvard Medical School. The gel pieces were rehydrated with 50 mM NH_4_HCO_3_ containing 12.5 µg/mL modified sequencing-grade trypsin (Promega) at 4°C for 45 min. Tryptic peptides were separated on a nano-scale C_18_ HPLC capillary column and analyzed after electrospray ionization in an LTQ linear ion-trap mass spectrometer (Thermo Fisher). The reference human proteome database was downloaded from UniProt (updated until March 2021). Peptide sequences and protein identities were assigned by matching the measured fragmentation patterns with protein or translated nucleotide databases using Sequest software. Peptides were required to be fully tryptic peptides with XCorr values of at least 1.5 for 1+ ions, 1.5 for 2+ ions, or 3.0 for 3+ ions. All data were manually inspected. Only proteins with ≥2 unique peptide matches were considered positively identified using a false discovery rate of <1% at peptide and protein levels (**Supplemental Table 2**).

### COVID Data Comparison With Coronascape

DS-affinity proteins were compared with currently available proteomic and transcriptomic data from SARS-CoV-2 infection compiled in the Coronascape database (as of 12/14/2020) (29–49). These data had been obtained with proteomics, phosphoproteomics, interactome, ubiquitome, and RNA-seq techniques. Up- and down-regulated proteins or genes were identified by comparing COVID-19 patients *vs*. healthy controls and cells infected *vs*. uninfected by SARS-CoV-2. Similarity searches were conducted between our data and the Coronascape database to identify DS-affinity proteins (or their corresponding genes) that are up- and/or down-regulated in the viral infection.

### Pathway and Process Enrichment Analysis

Pathways and processes enriched in the putative autoantigen-ome were analyzed with Metascape (29). The analysis was performed with various ontology sources, including KEGG Pathway, GO Biological Process, Reactome Gene Sets, Canonical Pathways, CORUM, TRRUST, and DiGenBase. All genes in the genome were used as the enrichment background. Terms with a p-value <0.01, a minimum count of 3, and an enrichment factor (ratio between the observed counts and the counts expected by chance) >1.5 were collected and grouped into clusters based on their membership similarities. The most statistically significant term within a cluster was chosen to represent the cluster. Pathway hierarchical clustering was obtained with ShinyGo (289).

### Protein-Protein Interaction Network Analysis

Protein-protein interactions among collections of DS-affinity proteins were analyzed by STRING (251), including both direct physical interaction and indirect functional associations. Interactions are derived from genomic context predictions, high-throughput lab experiments, co-expression, automated text mining, and previous knowledge in databases. Each interaction is annotated with a confidence score from 0 to 1, with 1 being the highest, indicating the likelihood of an interaction to be true. Only interactions with high confidence (a minimum score of 0.7) are shown in the figures.

### Literature Text Mining

Literature searches in Pubmed were performed for every DS-affinity protein identified in this study. Search keywords included the protein name, its gene symbol, alternative names and symbols, and the MeSH keyword “autoantibodies”. Only proteins with their specific autoantibodies reported in PubMed-listed journal articles were considered “confirmed” autoAgs in this study.

## Data Availability Statement

The original contributions presented in the study are included in the article or the **Supplementary Material**. Further inquiries can be directed to the corresponding authors.

## Author Contributions

JW directed the study, analyzed data, and wrote the manuscript. WZ performed some experiments and reviewed the manuscript. VR and MWR assisted in data analysis and manuscript preparation. MHR consulted on the study, analyzed data, and edited the manuscript. All authors contributed to the article and approved the submitted version.

## Funding

MHR acknowledges grants from the NIH/NCI (R21 CA251992 and R21 CA263262), a Cycle for Survival Equinox Innovation Grant, an Investigator Grant from the Neuroendocrine Tumor Research Foundation (NETRF), and support from the Farmer Family Foundation. Parts of the study were supported by the MSKCC NCI Cancer Center Support Grant (P30 CA008748). The funding bodies were not involved in the design of the study or the collection, analysis, or interpretation of data.

## Conflict of Interest

JW is the founder and Chief Scientific Officer of Curandis. MWR and VR are volunteers of Curandis. MHR is a member of the Scientific Advisory Boards of Trans-Hit Bio (Azenta Life Sciences), Proscia, and Universal DX.

The remaining author declares that the research was conducted in the absence of any commercial or financial relationships that could be construed as a potential conflict of interest.

## Publisher’s Note

All claims expressed in this article are solely those of the authors and do not necessarily represent those of their affiliated organizations, or those of the publisher, the editors and the reviewers. Any product that may be evaluated in this article, or claim that may be made by its manufacturer, is not guaranteed or endorsed by the publisher.
